# Protective immunity enhanced *Salmonella* vaccine vectors delivering *Helicobacter pylori* antigens reduce *H. pylori* stomach colonization in mice

**DOI:** 10.3389/fimmu.2022.1034683

**Published:** 2022-11-18

**Authors:** Amir Ghasemi, Shifeng Wang, Bikash Sahay, Jeffrey R. Abbott, Roy Curtiss

**Affiliations:** ^1^ Department of Infectious Diseases and Immunology, College of Veterinary Medicine, University of Florida, Gainesville, Florida, FL, United States; ^2^ Department of Comparative, Diagnostic and Population Medicine, University of Florida, Gainesville, FL, United States

**Keywords:** recombinant protein, cytokines, vaccine, protection, *Helicobacter pylori*, *Salmonella*

## Abstract

*Helicobacter pylori* is a major cause of gastric mucosal inflammation, peptic ulcers, and gastric cancer. Emerging antimicrobial-resistant *H. pylori* has hampered the effective eradication of frequent chronic infections. Moreover, a safe vaccine is highly demanded due to the absence of effective vaccines against *H. pylori*. In this study, we employed a new innovative Protective Immunity Enhanced *Salmonella* Vaccine (PIESV) vector strain to deliver and express multiple *H. pylori* antigen genes. Immunization of mice with our vaccine delivering the HpaA, Hp-NAP, UreA and UreB antigens, provided sterile protection against *H. pylori* SS1 infection in 7 out of 10 tested mice. In comparison to the control groups that had received PBS or a PIESV carrying an empty vector, immunized mice exhibited specific and significant cellular recall responses and antigen-specific serum IgG1, IgG2c, total IgG and gastric IgA antibody titers. In conclusion, an improved *S.* Typhimurium-based live vaccine delivering four antigens shows promise as a safe and effective vaccine against *H. pylori* infection.

## 1 Introduction

*H. pylori* is a Gram-negative bacterium infecting the stomach of more than half of the Earth’s human population and is the main cause of gastric pathologies including peptic ulcers, dyspepsia and gastric cancer (1, 2). Prevalence differs from 10 to >70% (3). *H. pylori* has been confirmed as a class I carcinogen by the World Health Organization (4). Gastric cancer is still the second leading cause of death by cancer worldwide (5). Eradication of *H. pylori* has been frequently considered as an efficient approach to cure peptic ulcer disease in addition to gastric cancer (6). No doubt, antibiotics are the first choice in treatment. However, *H. pylori* infections present numerous challenges to successful antimicrobial therapy, some of which are limited to *H. pylori* and others are experienced in the treatment of other infections. Challenges arise from the fact that *H. pylori* colonize the stomach where they are protected by a dense mucus layer and an acidic environment. Additionally, the stomach is continuously secreting acid and discharging its contents such that typical therapy would be diluted and washed out (7). The efficacy of several antimicrobials is greatly reduced at acidic pH and proper pH is needed for them to be effective. *H. pylori* can aquire resistant genotypes and become multi-drug resistant (MDR). Importantly, a recent study has shown that eradication of toxigenic *H. pylori* expressing VacA is not achievable using only antibiotics (8). In clinical practice, the quick emergence of resistance raised concerns about the correct management of this bacterial infection (9, 10). Thus, protective and therapeutic vaccines could be an alternative method for antibiotic treatment against *H. pylori* infection. The importance of CD4+ T cells in protective immunity against *H. pylori* has been broadly accepted (11). Oral immunization of recombinant *Salmonella* vectored vaccines could provoke classical Th1-type responses and also induce a significant mucosal SIgA response (12, 13) through transcytosis by micro-fold cells (14) or by direct antigen presentation by interstitial dendritic cells (15). It was reported that a considerable number of foreign antigens, synthesized and delivered by live attenuated *Salmonella* vector, protected animals against a diversity of pathogens including viruses, bacteria and parasites (16). Oral administration of a live attenuated *Salmonella* vector vaccine synthesizing and delivering *H. pylori* protective antigens was therefore early tested as an approach to eradicate *H. pylori* infections (17–26).

In this study, we employed recently developed much improved PIESVs that specifically synthesize heterologous antigens to enhance the induction of immune responses and protection against viruses and bacteria (27–29). The specific PIESV strain used in this study (29) has just been licensed by APHIS as the first genetically modified bacterial vectored vaccine in commercial distribution. Such mucosal PIESV delivery induces strong mucosal, systemic and cellular immunities against the targeted pathogen (30–35). Since *H. pylori* colonize and move through a mucosal body surface, the induction of mucosal immunity dependent on the production of secretory antibodies (SIgA/SIgM) as well as cellular immunity offers the first line of defense against infection with such pathogens (36–41). Injectable subunit recombinant protein vaccines are unable to induce such mucosal immunity in contrast to PIESVs that induce mucosal, systemic and cellular immunities that can block infections and preclude disease (27, 42–49). Here, we developed PIESVs using this most recently approved and licensed PIESV vector strain to synthesize and deliver *H. pylori* protective antigens to prevent *H. pylori* SS1 infection.

## 2. Materials and methods

### 2.1 Bacterial strains and growth conditions

Bacterial strains and plasmids used in this study are listed in **Table 1**. *Escherichia coli* and *S.* Typhimurium UK-1 derivative strains were routinely cultured in LB broth [19] or on LB agar at 37°C. *S*. Typhimurium UK-1 mutant strains were supplemented with 50 μg/ml of diaminopimelic acid (DAP), 0.05% arabinose, 0.1% mannose and 0.1% rhamnose when necessary for bacterial growth as described in previous work (29, 52–54). For animal experiments, *S.* Typhimurium χ12341 was cultured in LB broth with necessary supplements. Overnight cultures were diluted 1:100 into fresh medium and grown with aeration (200 rpm) to an optical density at 600 nm of ~0.85. Bacteria were then centrifuged at 5,000 × g for 15 min at room temperature and resuspended in buffered saline with 0.01% gelatin (BSG) (55). The *H. pylori* strain SS1 (a kind gift from Prof. James G. Fox, Massachusetts Institute of Technology) was grown on Brucella agar supplemented with 5% sheep blood, 25 μg/ml trimethoprim, 100 μg/ml vancomycin, 3.3 μg/ml polymyxin B, 50 μg/ml amphotericin B, 200 μg/ml bacitracinin and 10 μg/ml nalidixic acid in an anaerobic jar with a microaerophilic gas generating kit (BD, USA) for 5 days at 37°C.

**Table 1:** Plasmids and strains used in this study.

Strain or Plasmid	Genotype or relevant characteristics	Derivation or source
*E. coli*		
BL21(DE3)	F^-^ *ompT hsdS*_B_ (*r_B_^-^m_B_^-^*) *gal dcm* (DE3)	Novagen
χ6212	F^−^ λ^−^ φ80 Δ(*lacZYA-argF*) *endA1 recA1 hsdR17 deoR thi-1 glnV44 gyrA96 relA1* Δ*asdA4*	(50)
*Salmonella* Typhimurium χ12341	ΔP_murA25_::TT *araC* P_araBAD_ *murA* Δ*asdA27*::TT *araC* P_araBAD_ *c2* Δ(*wza-wcaM*)-*8* Δ*pmi-2426* Δ*relA197*::*araC* P_araBAD_ *lacI* TT Δ*recF126* Δ*sifA26* Δ*waaL46* Δ*pagL64*::TT *rhaRS* P_rhaBAD_ *waaL*	(29)
**Plasmid**		
pET28a+	Expressing vector, Kan+, pBR *ori*, T7 promoter	Novagen
pG8R111	Lysis vector, pBR *ori*, P_trc_, *araC* P_araBAD_ SD-GTG *murA*, weak SD-GTG *asdA*	(51)
pG8R114	Lysis vector with optimized *bla* SS, pBR *ori*, P_trc_ *araC* P_araBAD_ SD-GTG *murA*, weak SD-GTG *asdA*	(51)
pG8R60	The optimized *hpaA* gene of *H. pylori* fused with a C-terminal 6×His was cloned under control of the P_trc_ promoter of pG8R114.	This study
pG8R61	The optimized *ureA* gene of *H. pylori* fused with a C-terminal 6×His was cloned under control of the P_trc_ promoter of pG8R111.	This study
pG8R62	The optimized *cagA* gene of *H. pylori* fused with a C-terminal 6×His was cloned under control of the P_trc_ promoter of pG8R111.	This study
pG8R63	The optimized *babA* gene of *H. pylori* fused with a C-terminal 6×His was cloned under control of the P_trc_ promoter of pG8R114.	This study
pG8R64	The optimized *napA* gene *of H. pylori* fused with a C-terminal 6×His was cloned under control of the P_trc_ promoter of pG8R114.	This study
pG8R65	The optimized chimeric gene (*fliD*, *ureB, vacA*, *cagA*) gene of *H. pylori* fused with a C-terminal 6×His was cloned under control of the P_trc_ promoter of pG8R111.	This study
pG8R66	The optimized *ureB* gene of *H. pylori* fused with a C-terminal 6×His was cloned under control of the P_trc_ promoter of pG8R111.	This study
pG8R165	The optimized *hopM* gene of *H. pylori* fused with a C-terminal 6×His was cloned under control of the P_trc_ promoter of pG8R114.	This study
pG8R166 pG8R230	The optimized vacA gene of H. pylori fused with C-terminal 6xHis was cloned under control of the P_trc_ prompter in pG8R111. The optimized *ureA and* P_lpp_ *ureB* genes of *H. pylori* were fused together with a C-terminal 6×His and was cloned under control of the P_trc_ promoter of pG8R114.	This study This study
pG8R232	The optimized *napA* and P_lpp_ *ureA* genes of *H. pylori* were fused together with a C-terminal 6×His and was cloned under control of the P_trc_ promoter of pG8R114.	This study
pG8R233	The optimized *napA* and P_lpp_ *ureB* genes of *H. pylori* were fused together with a C-terminal 6×His and was cloned under control of the P_trc_ promoter of pG8R114.	This study
pG8R234	The optimized *napA* and P_lpp_ *ureA* genes of *H. pylori* were fused together with a C-terminal 6×His and was cloned under control of the P_trc_ promoter of pG8R114.	This study
pG8R235	The optimized *hpaA* and P_lpp_ *ureB* genes of *H. pylori* were fused together with a C-terminal 6×His and was cloned under control of the P_trc_ promoter of pG8R114.	This study
pG8R262	The optimized *napA* and P_lpp_ *hpaA* genes of *H. pylori* were fused together with a C-terminal 6×His and was cloned under control of the P_trc_ promoter of pG8R114.	This study
pG8R289	The *ureA* gene of *H. pylori* fused with a C-terminal 6×His cloned into the *Nco*I and XbaI sites in pET28a	This study
pG8R290	The *ureB* gene of *H. pylori* fused with a C-terminal 6×His cloned into the NcoI and XbaI sites in pET28a	This study
pG8R291	The *hpaA* gene of *H. pylori* fused with a C-terminal 6×His cloned into the NcoI and XbaI sites in pET28a	This study
pG8R292	The *napA* gene of *H. pylori* fused with a C-terminal 6×His cloned into the NcoI and XbaI sites in pET28a	This study

### 2.2 Plasmids and constructs

**Figure 1:**
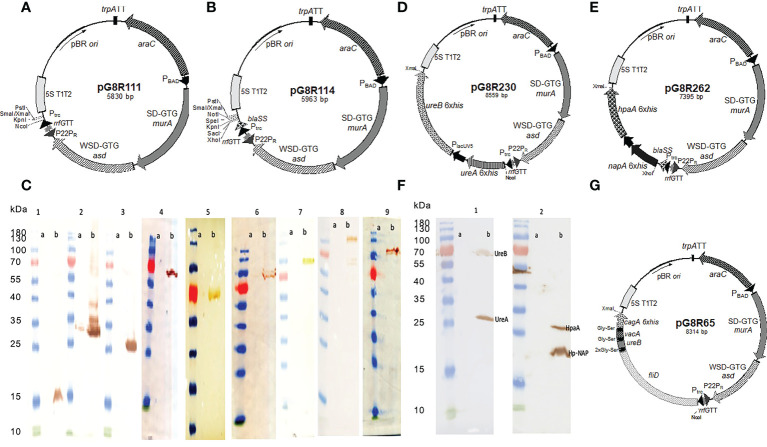
Synthesis of (*H*) *pylori* antigens in *S*. Typhimurium χ12341. Regulated delayed lysis plasmid vectors pG8R111 **(A)** and pG8R114 with improved *bla* SS **(B)**. **(C)** Synthesis of nine putative (*H*) *pylori* antigens in *S*. Typhimurium χ12341. (1) Hp-NAP (17.7 kDa), (2) HpaA (26.9 kDa), (3) UreA (27.5 kDa), (4) UreB (62.3 kDa), (5) HopM (74.6 kDa), (6) BabA (79.6 kDa), (7) Chimeric Protein (90 kDa), (8) CagA (131.8 kDa), (9) VacA(136.3 kDa). (a) Uninduced with IPTG and (b) 2 h after induction with IPTG compared to molecular mass markers. Anti-6xHis monoclonal antibody was used to detect each recombinant protein by western blotting. **(D).** Regulated delayed lysis plasmid vectors pG8R230 encoding optimized *ureA and* P_lpp_ *ureB* (*H*) *pylori* genes inserted into pG8R114 with expression controlled by P_trc_ promoter and pG8R262 **(E)** encoding optimized *napA* and P_lpp_ *hpaA* (*H*) *pylori* genes inserted into pG8R114 with expression controlled by P_trc_ promoter. **(F)** Successful simultaneous synthesis of four protective (*H*) *pylori* antigens using two different plasmids in *S*. Typhimurium χ12341. (1) UreA and UreB encoded on pG8R230 and (2) Hp-NAP and HpaA encoded on pG8R262. (a) Uninduced with IPTG and (b) 2 h after induction with IPTG compared to molecular mass markers. Anti-6xHis monoclonal antibody was used to detect each recombinant protein in western blotting. **(G)** Regulated delayed lysis plasmid vector pG8R65 encoding the *fliD-ureB-vacA-cagA* fusion derived from pG8R111.

The regulated delayed lysis vector pG8R111 (**Figure 1A**) for synthesis and delivery of antigens has a P_trc_-regulated synthesis of encoded protein antigens for delivery by cell lysis and *araC* P_araBAD_-regulated *murA* and *asdA* genes with GTG start codons to lessen translation efficiency. The pG8R111 has a weaker SD AAGGCAA to further reduce the production of AsdA. The P22 P_R_ located with opposite orientation to the transcription of the *araC* P_araBAD_ GTG-*murA* GTG-*asdA* genes is repressed by the C2 repressor made during the growth of χ12341 with arabinose. C2 concentration decreases due to cell division *in vivo* to cause P_R_-directed anti-sense mRNA synthesis to block translation of residual *asdA* and *murA* mRNA. Transcription terminators (TT) flank all plasmid domains for regulated lysis, replication, and gene expression so that expression in one domain does not interfere activities of adjacent domain. pG8R114 (**Figure 1B**) is derived from pG8R111 with a much-improved optimized β-lactmase signal sequence (56), the fusion of molecules to the *bla* SS in pG8R114 leads to delivery of molecules to the periplasm that results in increased production of outer membrane vesicles (OMVs) and releasing into the supernatant fluid surrounding PIESV cells that enhance induced immune responses (57, 58). We made constructs to synthesize nine putative protective *H. pylori* antigens according to the antigen gene sequences in *H. pylori* SS1 (2, 59–67) (**Table 1**). These constructs were evaluated for synthesis and delivery and protective immunity induction in mice. Antigens included VacA, CagA, UreB, UreA, HpaA, BabA, HopM, Hp-NAP and a chimeric antigen. To design the chimeric antigen with the most antigenic fragments of FliD, UreB, VacA and CagA, bioinformatic tools (65) were used to identify T-cell and B-cell epitopes. Sequences encoding FliD (1–600), UreB (327–385), VacA (744–805) and CagA (51–100) polypeptides were accessed from GenBank. To assist epitope exposure, flexible glycine-serine (GS) linkers were included between the gene segments. Sequences were codon-optimized to have a high-level expression in *S.* Typhimurium and GC contents adjusted to be closer to that for *Salmonella*. All genes were synthesized by Biomatik (Cambridge, Ontario, Canada). To detect the synthesis of recombinant proteins, a 6 His-tag sequence was added at the 3′ end of each gene before the stop codon. Based on the presence or absence of a signal peptide encoded in each antigen gene, antigens were divided into two groups. For those antigens without a signal peptide, the synthesized genes were cloned into pG8R111 whereas for those *H. pylori* antigens with signal sequences, these were removed, and the codon-optimized sequences were inserted into pG8R114 with fusion to the *bla* SS (**Table 1**). Considering the results obtained from the initial protection experiments (data not shown), four antigens were selected for further study. The codon-optimized *H. pylori* genes were fused with P_lpp_ in front of the second gene and then were cloned under control of the P_trc_ promoter of pG8R111 or pGR114 (**Table 1**) (**Figure 1**). Plasmids carrying genes were finally electroporated into χ12341. To obtain purified recombinant Hp-NAP, UreA, UreB and HpaA, sequences encoding each antigen were cloned into pET28a (+) (**Table 1**) using *Nco*I at N-terminal and *Xba*I at C-terminal sites. pET28a derivatives with inserts were electroporated into *E. coli* BL21(DE3) (**Table 1**) for synthesis and purification of gene products.

### 2.3 Determination of plasmid stability

Measurement of plasmid stability is described previously (55). Briefly, vaccine strains grown overnight (G0) were diluted 1:1000 into pre-warmed fully supplemented LB grown (permissive growth conditions) with aeration for 12 h at 37°C. This process was repeated for approximately 50 generations (G5) and the proportions of cells possessing the Asd^+^ plasmids were determined for each culture. The percentage of clones holding the plasmids from each culture was obtained by counting the colonies grown on LB agar without and with DAP. The ability of these clones to synthesize *H. pylori* antigens was also checked after 50 generations using western blotting.

### 2.4 Recombinant protein synthesis evaluated by SDS-PAGE and western blot analyses

*S.* Typhimurium strain χ12341 carrying recombinant plasmids (PIESV-Hp) or empty vectors (PIESV-empty vector) were cultured in LB containing 0.05% arabinose, 0.1% mannose and 0.1% rhamnose at 37°C. LB containing kanamycin 50 μg/ml was used to culture *E. coli* BL21 carrying recombinant plasmids as well. When the bacteria reached an OD600 of 0.6, 1 mM IPTG was added to the cultures to induce heterologous *H. pylori* protein syntheses. Protein samples from bacterial cultures were separated and analyzed by SDS-polyacrylamide gel electrophoresis with transfer to nitrocellulose membranes as previously described (68). Recombinant proteins were detected by anti-6xHis peroxidase (Roche, Basel, Switzerland) (1/40,000 dilution) for 2 h. *E. coli* BL21carrying pG8R289 (*ure*A-6xHis), pG8R290 (*ure*B-6xHis), pG8R291 (*hpaA*-6xHis) or pG8R292 (*napA*-6xHis) were grown overnight at 37°C in LB broth supplemented with 50 μg/ml kanamycin. The procedures for protein synthesis and purification have been described in our previous study (4). Prestained Protein Ladder, 10 to 180 kDa (Thermofisher, USA) was used as the protein marker.

### 2.5 Immunization of mice

All animal experiments were approved by the University of Florida Institutional Animal Care and Use Committee. Six-to eight-week-old SPF female C57BL/6 mice (n=10/group), were purchased from Charles River Laboratories (Wilmington, MA). Mice were acclimated for one week after arrival before starting the experiments.

Immunization procedures followed the previous description (4, 68, 69). Briefly, food and water were not given to mice for 6 h prior to the immunization and re-supplied 30 min later. Mice were orally immunized with 20 μl BSG containing 10^9^ CFU of each vaccine strain, combination of strains, or with 20 μl BSG alone as the negative control on day 0 and boosted on days 14 and 28. Blood samples were collected individually on days 0, 14, 28, 42 and 72 and serum collected individually for analysis of antibody responses. Mice (10 mice) were immunized with 10^9^ CFU of each PIESV synthesizing and delivering different antigens. A mixture of PIESV strains delivering pG8R111 and pG8R114 (PIESV-empty vectors) were used as controls.

### 2.6 Protection experiment

To assess whether the immunization of mice with *Salmonella* delivering *H. pylori* antigens was able to reduce bacterial burden of *H. pylori* in the stomachs of infected mice, mice were infected with 5×10^8^ *H. pylori* SS1 thrice at one day intervals two weeks after the last immunization. Four weeks post infection, mice were euthanized and their stomachs removed and *H. pylori* CFUs quantified. To gain CFUs, the quantitative bacterial culture of mouse stomach was used. Briefly, a half section of the stomach from each euthanized mouse was completely homogenized, serially diluted, and then plated on selective medium as described above.

### 2.7 Evaluation of serum and mucosal antibody responses by ELISA

An enzyme-linked immunosorbent assay (ELISA) was used to specifically evaluate serum total IgG, IgG1 and IgG2c titers in immunized mice. 96-well polystyrene plates (Nalge Nunc. Rochester, NY, USA) were coated with purified recombinant HpaA, UreA, UreB and Hp-NAP (5 mg/ml). After over-night incubation, the plates were washed three times with TBST buffer (Tris-buffered saline, pH 7.4, containing 0.05% Tween 20) and followed by blocking with 300 µl PBS containing 10% FBS for 2 h at 37°C. After adding serial dilutions of mouse sera to the plates, they were incubated for 2 h at room temperature and then washed with TBST. HRP-conjugated goat-anti-mouse IgG1, IgG2c or total IgG (Southernbiotech, Birmingham, AL, USA) were added to the wells and incubated for 90 min at 37°C. Following the last washing step, specific reactivity was calculated by the addition of 50 µl/well of the TMB substrate (Thermofisher, USA). The reaction was stopped by adding 15 µl of 2 M H_2_SO_4_. Next, the absorbance at 450 nm was measured. To define a cut-off value for the test, the mean OD plus three-fold SD of sera from mice immunized with BSG was calculated at a 1:100 dilution. ELISA titers were calculated as the reciprocal of the last serum dilution providing an OD higher than the cut-off (70). To assess stomach mucosal IgA production, the secretory IgA was extracted by incubation of mouse stomachs in PBS containing 5% non-fat dry milk, 4-(2-aminoethyl)-benzenesulphonyl fluorid (Calbiochem), 1 mg/ml 3.25mM aprotinin, 10 mM leupeptin (Sigma), and bestatin (Boehringer-Mannheim Biochemicals). After extensive vortexing and centrifugation (16,000 g for 10 min), the supernatants were used for the determination of antibody titers. An ELISA was used as described above.

### 2.8 T-cell activation cell and cytokine profiling

#### 2.8.1 Isolation of DCs from mouse bone marrow

C57BL/6 mice were euthanized, femurs were removed under sterile conditions, and then soaked in 70% alcohol for a minute. Both epiphyses were cut off with scissors, and the needle of a 1-mL syringe was inserted into the bone cavity to rinse the bone marrow out of the cavity into a sterile culture dish with RPMI 1640 supplemented with 2 mM L-glutamine, 100 U/ml penicillin, 100 µg/ml streptomycin and 10% heat-inactivated fetal bovine serum. The cell suspension in the dish was collected and counted. The cells suspended were distributed in plates at a density of 2×10^6^ cells/per plate. Subsequently, GM-CSF was added into the medium to a final concentration of 20 ng/mL. The cells were cultured at 37°C in an incubator containing 5% CO_2_. The culture medium was replaced 72 h later to remove the unattached cells and cell debris, afterwards GM-CSF was added to the fresh medium. On day 10, the semi-suspended cells and loosely attached cells were collected by gently pipetting the medium against the plate. The cells were pulsed with recombinant proteins (HpaA, Hp-NAP, UreA and UreB) and incubated in complete medium overnight before co-culturing with T cells.

#### 2.8.2 Antigen-specific T-cell assay

Mouse spleens were obtained from mice immunized with PIESV-Hp (*hpaA*, *napA*, *ureA*, *ureB*), PIESV-empty vector or PBS, at 45 days post the first immunization. T cells were isolated and counted using EasySep™ Mouse T Cell Isolation Kit (STEMCELL, Cambridge, MA.USA). T cells were then stained with CellTrace (Thermofisher, USA) to trace the propagation of T cells after re-stimulation by flow cytometry. After that, T cells and DCs already pulsed with recombinant proteins (HpaA, UreA, UreB and Hp-NAP) were co-cultured 10 to 1, (T cells to DCs, respectively) and incubated for 7 days. Supernatants were collected and cells were stained by antibodies against CD3, CD4, and CD8. Using Flow cytometer, T-cell propagation against each antigen in addition to different types of T cells were investigated.

Cytokine assay: The concentration of ten different cytokines including IFN-γ, TNF-α, IL-2, IL-4, IL-5, IL-6, IL-10, IL-17A, IL-22, and IL-23 were measured in both sera and supernatants described in the previous section. We used Multiplex Assays Using Luminex (Millipore-Sigma, USA) to evaluate the concentration of cytokines in the samples. In this method, the sample is added to a mixture of color-coded beads, pre-coated with different cytokine-specific capture antibodies. The antibodies finally bind to the interested cytokines. Specific detection antibodies of cytokines of interest conjugated with biotin are added and form an antibody-antigen sandwich. PE-conjugated streptavidin is then added. It attaches to the biotinylated detection antibodies. Polystyrene beads are read on a dual-laser flow-based detection set, using Luminex 200™. One laser categorizes the bead and defines the cytokine that is being detected. The second laser defines the greatness of the PE-derived signal, which is in direct quantity to the amount of cytokine bound.

### 2.9 Immunological responses in stomach of immunized mice after infection with *H. pylori*

#### 2.9.1 Spleen Single-cell Suspension Preparation and Flow Cytometry

To obtain further information about how the immune system responded in the immunized mice after infection with *H. pylori*, ten days after the challenge (day 55) mouse spleens were collected. Single-cell suspensions from spleens were prepared by pressing spleens through 100 μm cell strainers. Cell suspensions were washed in PBS and resuspended in red blood cell lysis buffer [155 mM NH_4_Cl; 10 mM KHCO_3_; 0.1 mM EDTA] for 8 min on ice. Cell suspensions were washed again with PBS before staining. Antibodies were purchased from eBioscience (CD8a, IFN-γ, IL-17A, CD16/32, and TNFα), BioLegend (GranzymeB, Aqua Live/Dead), or TONBO(CD3 and CD4). CD16/32 antibody was used to block nonspecific binding to Fc receptors before all surface staining. For cytokine staining, cells were stimulated with 500 ng/mL ionomycin and 50 ng/mL phorbol-12-myristate-13-acetate (PMA) for 4 h, and Brefeldin A (2 mg/mL) was added 2 h before cell harvest. Dead cells were discriminated by LIVE/DEAD Fixable Violet Dead Cell Stain Kit (Invitrogen). Sample acquisition was performed on FACSCantoII and analyzed with FlowJo (version 10.2, Tree Star).

#### 2.9.2 Quantitative real-time PCR analysis

The expression of CXCL1, CXCL2, CXCL5, Reg3a, Reg3b, Reg3d and Reg3g were investigated using qPCR (71, 72). Briefly, RNA extracted from mouse stomach biopsy specimens obtained ten days after infection were reverse-transcribed to cDNA by PrimeScriptTM RT reagent Kit (ThermoFisher). Real-time PCR was performed on the IQ5 (Bio-Rad) with PowerUpTM SYBERTM Green Master Mix (appliedbiosystems, USA) according to the manufacturer’s specifications. β-actin was used for normalizing the expression. The relative gene expression was expressed as fold change calculated by the ΔΔCt method (73).

### 2.10 Histochemistry

Mouse stomachs were obtained 75 days post the first immunization. Stomach tissues were fixed in 10% neutral buffered formalin for at least 24 h. The trimmed tissues were processed, paraffin-embedded, sectioned at 5 μm, and stained with hematoxylin and eosin. Selected sections of stomach tissues were also evaluated with Warthin-Starry and Gram stains to identify bacteria. The pathologist was blinded as to the experimental groups and treatments of the study. Histologic samples of both glandular and squamous portions of the stomach were examined and evaluated and scored based on intensity 0 to 3 for criteria including mucosal inflammation and type, submucosal inflammation and type, non-*H. pylori* bacteria, mucosal ulceration, and hyperkeratosis of squamous stomach.

### 2.11 Statistical analysis

SPSS computer software, version 17.0. was used to do statistical analysis. The statistical differences between two groups were studied by t-test and results among several groups were obtained by one factor analysis of variance (ANOVA) and Tukey’s *post hoc* test. The statistical border for accepting significance was *P*< 0.05. Data were envisaged using the GraphPad Prism 7 program.

## 3. Results

### 3.1 *Salmonella* Strain χ12341 and Plasmid encoding *H. pylori* antigens

The construction and properties of χ12341 have been recently described (29). Three sugars, arabinose, mannose and rhamnose which are not present in animal tissues, were used to regulate the virulence trait. The ΔPmurA::TT *araC* P_araBAD_ *murA* and *ΔasdA*::TT *araC* P_araBAD_ *c2* mutations regulate synthesis of diaminopimelic acid and muramic acid, two essential constituents of peptidoglycan, which enable PIESV lysis in the absence of DAP and inability to synthesize the MurA enzyme without arabinose *in vivo* (53). The *Δpmi ΔwaaL ΔpagL*::TT *rhaRS* P_rhaBAD_ *waaL* mutations collectively provide regulated delayed attenuation and cause cessation in the synthesis of LPS O-antigen in the absence of the sugars mannose and rhamnose *in vivo* (52, 74, 75). The deletion Δ(*wza-wcaM*) enhances complete lysis, blocks the synthesis of cell surface polymers to prevent biofilm formation and enhances induced immunities (76). The *ΔrelA*::*araC* P_araBAD_ *lacI* TT mutation confers the regulated delayed antigen synthesis phenotype that enhances PIESV colonization and induction of immune responses by the arabinose-dependent regulated LacI synthesis so that P_trc_-regulated genes on plasmid vectors are gradually expressed as LacI is diluted at each cell division during *in vivo* growth (77–79). The Δ*recF* mutation decreases inter- and intra-plasmidic recombination to stabilize PIESVs (80). The Δ*sifA* mutation enables PIESV escape from the *Salmonella*-containing vesicles (SCVs) to cause PIESV cells to lyse in the cytosol (81, 82). This enables delivery of protective antigens to the proteasome for class I presentation to generate CD8-, CD17- and NKT-dependent immunities.

Codon-optimized sequences encoding all nine *H. pylori* antigens were successfully inserted into the plasmid vectors pG8R111 or pG8R114 (**Figures 1A, B**) to yield the plasmids listed in **Table 1**. The synthesis of each antigen was confirmed by western blot using a monoclonal antibody against the 6His-tag (**Figure 1C**) to yield the plasmids listed in **Table 1**. Results of the plasmid stability experiment showed that all recombinant plasmids encoding antigens except HopM were stable in the *Salmonella* vaccine strain. However, only 30% of *Salmonella* cells lost the plasmid specifying HopM synthesis after 50 generations of growth under permissive conditions. Based on results obtained from initial protection experiments, UreA+UreB (pG8R230) and HpaA+Hp-NAP (pG8R262) (**Figures 1D, E**), the set of antigens providing considerable protection, were selected to be expressed by pG8R111 and pG8R114, respectively. χ 12341(pG8R230) and χ 12341(pG8R262) specified synthesis of the four encoded antigens after IPTG induction (**Figure 1F**). We also designed and expressed a chimeric antigen including protective parts of four four putative antigenis (FliD, VacA, CagA and UreB) of *H. pylori* using pG8R111 in PIESV χ 12341 (**Figure 1G**).

### 3.2. Immunization of mice with *Salmonella* carrying pG8R230(UreA+UreB) and pG8R262(HpaA+HpNAP) induces significant protection against *H. pylori* SS1 challenge with strong specific humoral and mucosal immune responses

**Figure 2:**
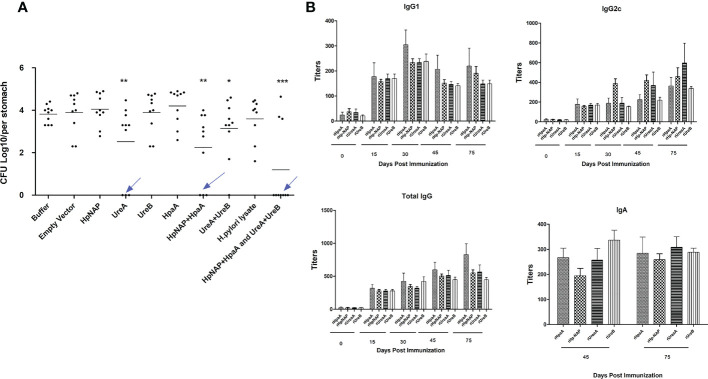
**(A)** Protection against (*H*) *pylori* SS1 challenge infection following oral vaccination of mice with individual and combinations of χ12341 strains harboring plasmids listed in **Table 1** synthesizing and delivering specified (*H*) *pylori* antigens. **(B)** Specific antibody responses in immunized animals. Kinetics of specific antibody responses after oral immunization with a cocktail vaccine of χ12341(pG8R230) and χ12341(pG8R262) delivering UreA and UreB and NapA and HpaA, respectively. Mice were bled (submandibular bleeding method) on the indicated days, and specific IgG1 and IgG2c and total IgG antibody titers against recombinant UreA, UreB, NapA and HpaA evaluated by ELISA. Titer values represent the mean ± SD of sera from three analyses of five animals each. Stomach suspensions were obtained 45 days post first immunizationand specific IgA titers were analyzed by ELISA.

To determine whether immunization with vaccine candidates lowers the bacterial burden in the stomachs of infected mice, we ascertained CFUs of *H. pylori* using quantitative bacterial culture procedures. Immunized mice were infected with *H. pylori* SS1 two weeks after the last immunization. Then, four weeks after challenge, the stomachs of euthanized mice were removed, minced, homogenized, serially diluted and then plated on the selective agar medium. As shown in **Table 2**; **Figure 2A**, higher levels of protection were observed in mice immunized with the *Salmonella* vaccine PIESV-Hp strains carrying pG8R230(UreA+UreB) and pG8R262(HpaA+HpNAP). Notably, seven out of 10 immunized mice in this group showed sterile protection and the other three mice showed a significant reduction in a load of bacteria compared to mice receiving BSG or empty vector (**Figure 2A**).

**Table 2:** Colonization of stomach by *H. pylori* in immunized mice.

Group	Antigens	Log10 CFU of *H. pylori*	Group	Antigens	Log10 CFU of *H. pylori*
1	HpaA	4.2	13	Hp-NAP + HpaA	1.9**
2	Hp-NAP	4	14	HpaA + UreB	1.8**
3	UreA	2.52*	15	UreB + VacA	4
4	UreB	3.9	16	VacA + UreA	1.9**
5	UreB + UreA	1.8**	17	Hp-NAP + UreB	1.63**
6	VacA, + Hp-NAP	1.9**	18	UreA + HopM	3.3
7	UreA + HpaA	1.4***	19	HopM + Hp-NAP	4.3
8	HpaA + BabA	3.34	20	*H. pylori* lysate	2.8*
9	CagA + VacA	2.31*	21	BSG	3.6
10	Chimeric (FliD, UreB, VacA, CagA)	2.3*	22	Empty vector	3.7
11	BabA + HopM	3.8	23	*UreA + UreB HpaA + Hp-NAP	1.19***
12	HopM + CagA	2.45			

*P < 0.05; **P < 0.01; ***P < 0.001 compared with PBS and Empty vector

Groups refer to immunization of groups of ten mice with χ12341 containing the plasmids listed in **Table 1** encoding the H. pylori antigens specified.

To study the humoral response against antigens in mice immunized with PIESV vector strains delivering four antigens, sera were obtained at several time points over three months following initial immunization. Immunization of mice induced strong and specific immunoglobulin G (IgG) responses against each component of the cocktail vaccine including pG8R230(UreA+UreB) and pG8R262(HpaA+HpNAP), in which IgG2c (Th1-related isotypes) titers were usually slightly higher than those of the IgG1 subtype (**Figure 2B**). The total IgG and IgG2c titers against each antigen started to increase during the second week after the first immunization and peaked after 75 days. However, IgG1 titers peaked at 30 days post first immunization and titers fell after 75 days. These results indicated that immunization of mice with *Salmonella* vaccine vectors carrying pG8R230(UreA+UreB) and pG8R262(HpaA+HpNAP) elicit a Th1-biased immune response. To evaluate whether immunization with PIESV strains delivering these four antigens also provoked mucosal immune responses, gastric IgA production was evaluated for each antigen in stomachs of immunized mice. As shown in **Figure 2B**, the immunized mice with the cocktail of four antigens significantly augmented gastric mucosal IgA titers against each component of the cocktail vaccine. These findings indicate that immunization of mice with a combination of pG8R230(UreA+UreB) and pG8R262(HpaA+HpNAP) synthesized and orally delivered by two PIESV vector strains provokes both specific systemic and mucosal humoral immune responses.

### 3.3. Immunization of mice with *Salmonella* carrying pG8R230(UreA+UreB) and pG8R262(HpaA+HpNAP) induces mixed Th1-, Th2-, and Th17-type immune responses

#### 3.3.1 T-cell propagation

**Figure 3:**
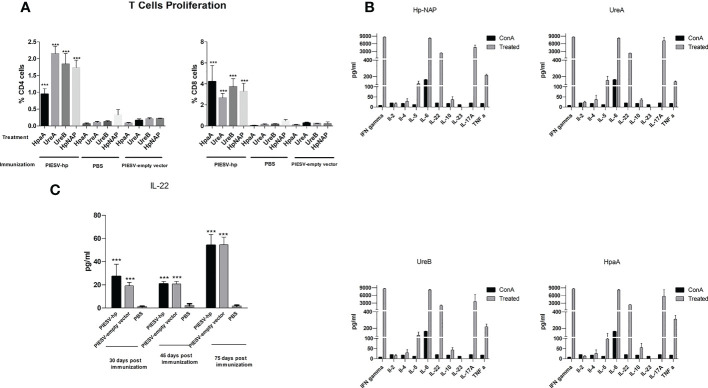
T cell responses **(A)** Proliferative responses of T cells from mice immunized with the χ12341(pG8R230) and χ12341(pG8R262) mixture, χ12341(pG8R111) = PIESV-empty vector) and PBS. T cells from immunized mice were isolated 45 days post first immunization and co-cultured with dendritic cells treated with 20 µg/ml recombinant NapA, HpaA, UreA and UreB and the proliferative responses were subsequently assayed by CellTrace. T cells were also stained with proper antibodies to be analyzed for the proliferation of CD4^+^ and CD8^+^ T cells by flow cytometry. **(B)** Cytokine production by T cells from mice immunized with the mixture of χ12341(pG8R230) and χ12341(pG8R262). T cells were obtained 45 days post first immunization from immunized mice and co-cultured with dendritic cells already treated with 20 µg/ml recombinant NapA, HpaA, UreA and UreB. Cytokine concentrations in culture supernatants were measured by multiplex ELISA. The data are the mean ± SD of five individual mice from each group. **(C)**. Level of IL-22 increased in mice immunized with the PIESV-Hp mixture. Sera obtained 30, 45 and 75 days after first immunization of mice with PIESV-Hp, PIESV-empty vectors and PBS were analyzed for the presence of IL-22. ***P < 0.001.

To find out the dominant subset of T cells in the spleen of immunized mice and assess whether such T cells could propagate after re-stimulation with related antigens *in vitro*, T cells were co-cultured with dendritic cells pulsed with each component of a cocktail vaccine composed of recombinant UreA, UreB, HpA and HpNAP proteins. The percentage of CD4+ and CD8+ cells in the immunized group were significantly increased compared with PBS or PIESV-empty vector immunized mice (**Figure 3A**). Additionally, CD8+ cells propagated more than CD4+ cells showing a dominant cytotoxic response in immunized mice in response to each antigen (**Figure 3A**).

#### 3.3.2. Cytokine production

Since cytokines secreted by activated T cells are indicators of the type of Th responses, we also measured the amounts of ten different cytokines in supernatants obtained in T-cell propagation experiments. Cytokine production by the re-stimulated T cells was determined by Multiplex ELISA. In comparison to the controls, T cells of mice immunized with the combination of pG8R230(UreA+UreB) and pG8R262(HpaA+HpNAP) synthesized and delivered by two PIESV vector strains secreted significantly higher amounts of IFN-γ, IL-5, IL-6, IL-22, IL-17A and TNF-α in response to each recombinant protein used for immunization (**Figure 3B**). We could also detect small amounts of IL-2, IL-4 and IL-10 but not IL-23 secreted against each antigen after re-stimulation with related antigens. These results suggest that immunization of mice with a combination of Hp-NAP, UreA, UreB and HpaA synthesized by the PIESV strains induces a mix of Th1, Th2, and Th17 responses. Further study using sera obtained from immunized mice showed that the level of circulating IL-22 cytokine increased following immunization compared to the non-immunized control groups (**Figure 3C**).

### 3.4 Immunized mice increase IFN-γ+ CD4 T and CXCL2 after infection with *H. pylori* SS1

**Figure 4:**
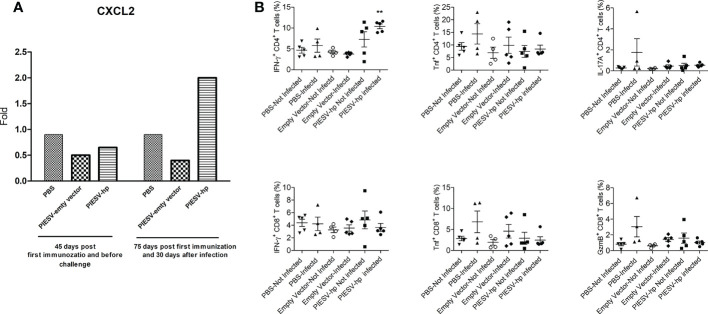
**(A)** CXCL2 is increased in stomach of (*H*) *pylori* SS1-infected mice immunized with PIESV-Hp mixture. CXCL2 mRNA synthesis in stomach of (*H*) *pylori* SS1-infected mice immunized with PIESV-Hp, and PIESV (empty vector) and PBS were compared (n = 10). **(B)** PIESV-Hp mixture immunized mice showing increases in IFN-γ+ CD4 T after challenge infection with (*H*) *pylori* SS1. Fifty-five days after the first immunization, mouse spleens were collected. Single-cell suspensions from spleens were isolated and then stained with proper antibodies to be analyzed by flow cytometry. **P < 0.01.

Antimicrobial peptide plays a important role in immune responses to *H. pylori* (83, 84). To obtain a deeper understanding of the roles of antimicrobial peptides and immunological markers in the protection provided in immunized mice, ten days after infecting the tested mice with *H. pylori* SS1, mouse stomachs and spleens were obtained. Reg3a, Reg3b, CXCL1, CXCL2, and CXCL5 in stomach tissues were investigated by qPCR. These chemokines and antimicrobial peptides were selected since they have been shown to work in correlation with IL-17 (85, 86). Spleen samples were further analyzed for T-cell markers and cytokines. In the stomachs of immunized mice after infection with *H. pylori* SS1, the expression of CXCL2 increased two-fold while the expression of the remaining genes did not change (**Figure 4A**).

In response to *H. pylori* SS1 infection, IFN-γ+ CD4 T cells increased in the spleens of immunized mice compared to the controls. Additionally, no significant changes in TNF+ or IL-17A+ CD4 T cells in the spleen for any test groups were seen. There were also no significant changes in IFN-γ+, TNF+ or GranzymeB+ CD8 T cells in the spleen for any test group (**Figure 4B**).

### 3.5 Histopathology

The stomach tissues were evaluated and scored based on severity 0 to 3 for criteria including mucosal inflammation and type, submucosal inflammation and type, presence of non-*H. pylori* bacteria, mucosal ulceration, and hyperkeratosis of squamous portions of the stomach. The scale 0-3 refers to 0=normal, 1=mild, 2=moderate, 3=severe. The inflammation ranged from nominal to severe both in the mucosa and submucosa of both the glandular and squamous portions of the stomach and consisted of lymphocytes, plasma cells with or without smaller numbers of neutrophils. Mice in control group had large amounts of hyperkeratosis in the squamous portions of the stomach which are often associated with moderate to large numbers of large, Gram-positive, rod-shaped bacteria on the surface or within the laminated keratin. The hyperkeratosis and associated bacteria are suggestive of hyporexia or anorexia for a relatively prolonged period of at least 2 days. In anorectic rodents, the build-up of excess keratin is presumably caused by reduced mechanical removal by the passage of food (87). The hyperkeratosis was not seen in a single area as suggested at the limiting ridge, but throughout all of the squamous lined areas of the forestomach.

**Figure 5:**
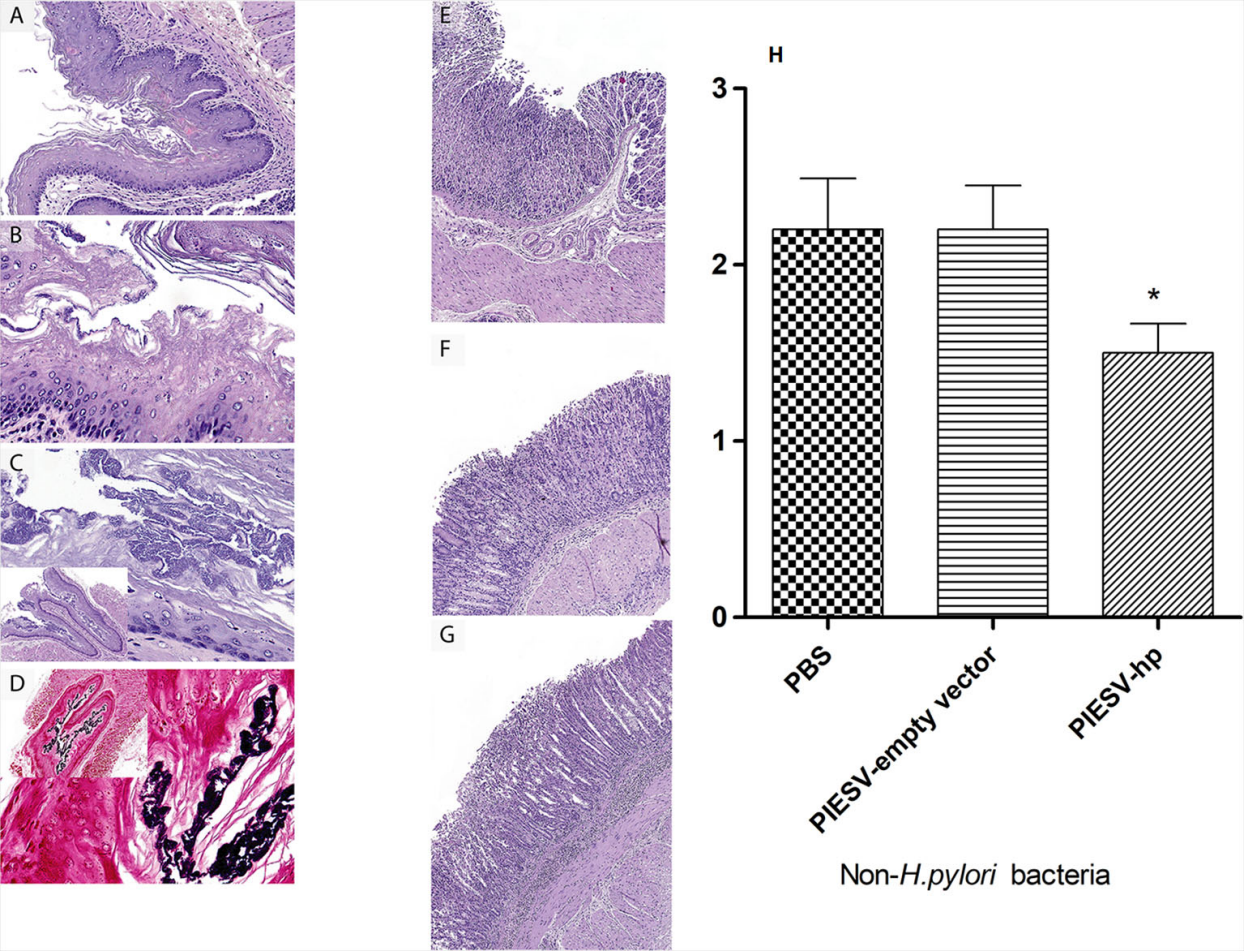
Immunization with the PIESV-Hp mixture reduces inflammation and the number of Gram-positive rod-shaped bacteria in the mouse stomach. Mice immunized with the PIESV-Hp mixture, the PIESV-empty vector and PBS were infected with (*H*) *pylori* SS1 two weeks post last immunization. After thirty days, mice were sacrificed, and stomach tissues were obtained for H&E staining. **(A)** Squamous portion of stomach of mice immunized with the PIESV-Hp mixture showed moderate amounts of hyperkeratosis. **(B)** Abundant hyperkeratosis with moderate numbers of large rod-shaped bacteria (Gram stain) associated with the keratin layer in mice immunized with PIESV-empty vector. **(C)** Same as B but with much larger populations of gram-positive bacteria in the keratin layer of mice immunized with PIESV empty vector. **(D)** Same as C but in mice immunized with PBS. Glandular portion of the stomach with **(E)** mild, **(F)** moderate, and **(G)** severe mucosal and submucosal inflammation in mice immunized with the PIESV-Hp mixture (E & F) or the PIESV empty vector or PBS **(G)**. **(H)** Reduction in the load of rod-shaped bacteria in immunized mice based of relative frequency scores of 0 (absent), 1 (low), 2 (moderate) and 3 (high). *P*<0.05.

However, significant weight loss was not seen in any group of mice. Overall, the mice receiving the PIESV-empty vector had relatively higher inflammation and numbers of non-*H. pylori* bacteria (gram-positive rod-shaped bacilli) compared to immunized mice. These findings might suggest the role of other gram-positive bacteria as well as *H. pylori* infection in the development of stomach complications. Additionally, these findings demonstrated that the vaccine candidate not only protects against *H. pylori* SS1 but also against other non-*H. pylori* flora, which may have a role in stomach complications (**Figure 5**).

## 4 Discussion

*Salmonella* has been extensively investigated for delivering recombinant protective antigens and DNA vaccine vectors due to its capability to be delivered mucosally, thereby providing needle-free immunization especially against *H. pylori* (17, 18, 27, 88). Although these vaccines were protective in animal models, they were less effective in humans (17, 21, 89, 90). Therfore, there is a need for an efficient *Salmonella* to synthesize and deliver protective antigens inside the host to immunize all armes of immune systems efficiently. Live attenuated bacterial vaccines preferably comprise strains that possess two or more stable attenuating mutations dispersed in the bacteria chromosome and plasmids (without antibiotic-resistant markers) into which genes encoding heterologous antigens from microbial pathogens can be introduced. The attenuating mutations should be in genes encoding essential components of bacterial cell structures or biosynthetic pathways for crucial nutrients that are not freely available in any environment that the PIESV may reside in the immunized animal host or outside of the laboratory (Clark-Curtiss and 51).

In this study, we employed a recently much improved PIESV vector strain with superior attributes compared to previously used recombinant attenuated *Salmonella* vaccine vector strains used by others or by us. Based on the succeed of *Salmonlla-C. perfringens* vaccine development (29), this PIESV vector strain χ12341 was thus used to deliver and synthesize nine recombinant protective antigens of *H. pylori*. Mice were immunized with each construct alone or a combination of them. Upon completion of these survey studies, we investigated PIESV constructs delivering combinations of two antigens (**Table 1**) to determine those providing the most favorable protection. Our findings showed that immunization of mice with pG8R230(UreA+UreB) and pG8R262(HpaA+HpNAP) provided the most significant level of protection against *H. pylori* SS1 infection. *H. pylori* strains display a high level of genetic heterogeneity (91). However, efforts to find a safe vaccine against *H. pylori* have resulted in using several protective antigens that are very conserved in *H. pylori* strains. To form a neutral microenvironment around the bacterium in the acidic environment of the stomach, *H. pylori* needs to produce large amounts of urease which is formed by two subunits, UreA and UreB (92), which have been broadly used as candidate antigens for the development of vaccines against *H. pylori* infection (93). HpaA has been identified on the surface of *H. pylori* and is highly conserved among *H. pylori* strains (94). Another well conserved antigen is Hp-NAP that is essential for both pathogenesis and immunity (59). Multivalent vaccines may stimulate more efficient and full protection against *H. pylori* infection. In designing multivalent vaccines, the possible interaction of components with each other should be given special attention such as synergistic and antagonistic effects or competition between components and even epitope suppression, resulting in an inappropriate immune response (93). Importantly, in this study, we observed that combination of UreA, UreB, HpaA and HpNAP when synthesized and delivered by a PIESV vector strain could provide synergistic effects. These results not only emphasize the efficacy of our cocktail vaccine to provide sterile protection but also show the strong applicability of PIESVs, especially the improved plasmids used in this system. To unravel the mechanism of protection provided by our cocktail vaccine, the humoral and cellular immune responses induced by immunization were evaluated and compared to mice immunized with the PIESV-empty vector strain or with PBS.

Though the detailed mechanism by which immunization protects against *H. pylori* remains to be clarified, the vital role of cell-mediated immunity has been well-known with evidence demonstrating that protection against *H. pylori* infection can be obtained even in the lack of B cells (95). In this regard, immunological eradication of *H. pylori* usually is contingent on Th1-type responses and IFN-γ, the Th1 cytokine, playing a main role in the control of *H. pylori* infection by activating macrophages and changing antibody responses toward protective IgG2c in C57BL/6 mice (96). Our data show very high levels of Th1 (IFN-γ and IL-12) and Th17 (IL-17) cytokines during recall responses of T cells from mice immunized with our cocktail vaccine including pG8R230(UreA+UreB) and pG8R262(HpaA+HpNAP). Consistent with the proliferative response, T cells from mice immunized with our vaccine, produced higher levels of IFN-γ in response to each component of the cocktail than mice that received PBS or a mixture of the empty vector PIESV strains. In addition, T cells of immunized mice produced high levels of IL-6 when re-exposed to the immunizing antigens. IL-6 is deemed one of the mediators directing the transition between innate and adaptive immunity. Its production is mostly targeted by the PAMP-mediated TLR signaling cascade and as a result, IL-6 gene-deficient mice display impaired defense against some bacterial infections (97). Thus, it is possible that this cytokine plays a key role in providing immunity against *H. pylori* infection. Besides, Th17 cells and their effector cytokines seem to mediate immunity against numerous infections, mainly those caused by extracellular pathogens such as *H. pylori* (98). Th17 immune responses include neutrophil recruitment, secretion of antimicrobial peptides, and Th1-mediated protection driven by IL-17 (99, 100). In our study, a significant amount of IL-17 was measured after the re-stimulation of T cells with each recombinant antigen in the cocktail vaccine. Our finding showed that the level of circulating IL-22 cytokine spiked after immunization with the PIESV-Hp mixture and the PIESV-empty vectorcompared to the non-immunized control group only receiving PBS. It seems that *Salmonella* vaccines are able to induce the production of circulating of IL-22 that has been shown to play an important role in protecting the gut tissue integrity and enhance disease lessening through chronic *Salmonella* Infection (101).

To gain insight further into the underlying mechanism, we evaluated the kinetics of antibody production against each component of the vaccine and found that both antigen-specific IgG1 and IgG2c antibodies were induced. Notably, the IgG1/IgG2c ratio also showed that the response elicited in immunized mice had a strong Th1 bias. Since IFN-γ and IgG2c are markers of the Th1 type of immune response in C57BL/6 mice, and synthesis of IgG1 can be affected by Th2 clones, seeing the results obtained from cytokine assays and these findings, one might conclude that the immune responses elicited by the vaccine was of a mixed Th1/Th17 type (96). However, our cytokine detection was limited to the serum and spleen such that it might not reflect the levels of cytokines produced in the stomach. Further research to evaluate the cytokine production in the stomach with RNA-seq and/or flow cytometry will provide a deeper insight about the dynamics and function of immune cell populations and cytokine production in the stomach.

We then measured the mucosal immune responses produced by immunization. With that goal, the gastric IgA production and immune-mediated inflammation were evaluated in immunized mice. Compared to control immunized mice, immunized mice elicited significant levels of each antigen-specific gastric IgA. These findings indicate that immunization with PIESV strains delivering heterologous antigens efficiently stimulated gastric mucosal immune responses.

We also investigated the responses of other components of the immune system in immunized mice against infection by *H. pylori*. Thus synthesis of several antimicrobial peptides and chemokines were assessed. Our results only showed that production of CXCL2 is significantly upregulated in the stomach of immunized mice. CXCL2 is also a mighty neutrophil chemoattractant and is involved in numerous immune responses comprising wound healing, cancer metastasis, and angiogenesis (102). This result shows that our vaccine is capable to induce recall innate as well as acquired immunity. Systemic immune responses were also studied in immunized mice. Although in proliferation assays, CD8+ T cells were propagated more than CD4+ T cells in response to re-stimulation, the number of CD4+ T cells but not CD8+ T cells expressing IFN-γ increased after infection with *H. pylori* SS1 in the spleen of immunized mice. This supports the IgG titer data that indicate that systemic immunity was skewed towards a Th1 response after immunization. However, local immunity in the stomach tissues may be more dependent on CD8+ T cells. Further investigation is therefore needed to clarify the contribution of CD4 versus CD8 T cells in the stomach tissue.

The results of histopathology measurements showed an increased number of gram-positive rod-shaped bacteria and hyperkeratosis resulting in excessive pressure, inflammation or irritation to the stomach mucosa of control groups compared to the immunized group. Although we did not verify the species of this population, this finding shows the role of *H. pylori* infection in increasing the number of other gram-positive bacilli which might have a role in worsening the stomach complications caused by *H. pylori* infection. In this regard, it has been indicated that gastric colonization by non-*H. pylori* bacteria, such as Bacteroides, Actinobacteria, Firmicutes, Fusobacteria, and Proteobacteria, could influence the risk for gastric cancer (103–107). Notably, our vaccine not only provided sterile protection in seven out of 10 of the immunized mice but it also caused a reduction in the titer of a gram-positive rod-shaped bacterium.

To sum up, a *S.* Typhimurium-based live vaccine including the delivery of four protective antigens of *H. pylori* confers significantly high protection against *H. pylori* infection in mice. Additionally, immunological studies showed the induction of both specific acquired immune responses against each antigen and the innate immunity responses.

## Data availability statement

The datasets presented in this study can be found in online repositories. The names of the repository/repositories and accession number(s) can be found in the article/**Supplementary Material**.

## Ethics statement

The animal study was reviewed and approved by University of Florida Institutional Animal Care and Use Committee.

## Author contributions

The experiments were conceived and designed by AG and RC and performed by AG, SW, BS, and JA. The data were analyzed by AG and RC. The manuscript was written by AG, SW, and RC. All authors contributed to the article and approved the submitted version.

## Funding

This work was supported partially by Pilot Award (P0109858) (AG) from the Clinical and Translation Sciences Institute (CTSI) at the University of Florida and by R01 AI60557 (RC) and R21 AI126172 (SW) grants from the (NIH).

## Conflict of interest

The authors declare that the research was conducted in the absence of any commercial or financial relationships that could be construed as a potential conflict of interest.

## Publisher’s note

All claims expressed in this article are solely those of the authors and do not necessarily represent those of their affiliated organizations, or those of the publisher, the editors and the reviewers. Any product that may be evaluated in this article, or claim that may be made by its manufacturer, is not guaranteed or endorsed by the publisher.
