# Supplementary material for: Protective immunity enhanced Salmonella vaccine vectors delivering Helicobacter pylori antigens reduce H. pylori stomach colonization in mice
Source: Front Immunol. 2022 Nov 18;13:1034683. doi: 10.3389/fimmu.2022.1034683 (PMC9716130; doi:10.3389/fimmu.2022.1034683)
Supplement: Supplementary file 1 [file DataSheet_1.docx]

**1-Hop E–**

**ACCESSION EF635415**

**2-HopM or Vac31**

**ACCESSION FM991728.1**

**3-HpaA or Vac 40**

**ACCESSION CP007605.1**

**4-UreA** urease subunit alpha

**GenBank: AIHW01000031.1**

**5-VacA**

**ACCESSION AY737319**

**6-CagA**

**ACCESSION DQ091000**

**7-neutrophil-activating protein**

**(NAP)**

**ACCESSION AB649170.1**

**8-BabA1**

**ACCESSION GQ272327**

**9-UreB**

**ACCESSION AY714224**

**10-FliD**

**ACCESSION U82981**

**11. *H. pylori* SS1**

**NZ_CP009260**
